# Disastrous Times

**Published:** 1874-01

**Authors:** 


HALL'S

### JOURNAL OF HEALTH

AND

MISCELLANY.

VOL. XXI.—JANUARY, 1874.—NO. I.

### DISASTROUS TIMES.

*How to help the Poor and not be made a penny the poorer.*

*How the Poor may be fed abundantly and healthfully at one quarter the present cost.*

*How thousands of the unemployed can themselves obtain work and comfortable places in twenty-four hours.*

THE poor are our brothers and sisters, and they ought to and must be helped in these evil days to them, when they would gladly work if they had work to do. Many are famishing for food and shivering for shelter in their scanty garments. How the full-handed, the well-to-do and the rich can help them and meet their necessities without being a penny the poorer, when the beautiful Spring-time comes, with its warmth and flowers, and its hopeful promise of a bounteous product in the harvest time, is well worth finding out. Said a mechanic to me, only yesterday, "Doctor, I went to my wife last week and proposed to her to sell our carriage and horses, not because there was any pecuniary necessity for it, but that it did not look humane for me to be a riding in this style when so many worthy people were in destitution, and who might, in their privations, bitterly complain of the great difference in their lot in life; and, like the noble wife that she is, she said, without a moment's hesitation, 'So we will; we can walk, as we used to do when we began housekeeping; and our daughters can walk too—it will do them good—while the keep of the horses, and the wages of the coachman, and the interest of the equipage can all go to the purchase of necessaries at wholesale, and we can find out ourselves where they are most needed and deserved.'" There, reader, the wife of a mechanic, who had climbed to fortune from the very bottom of the ladder of day's work by industry, energy and unconquerable determination, to live always within his means, has set an example as wise as it is queenly, which solves at once the difficulty of the times, and the news of it ought to reach every hamlet and every palatial mansion in the land with telegraphic speed, for it embodies a lesson, a principle which the well-to-do ought to begin to carry out practically on the instant. *Do without all we can* until these emergencies are passed. Let nothing be purchased—not a ribbon, or a segar, until the question is deliberately considered, and, in the light of humanity, conscientiously answered, "Can't I do without it; can't I make this dress and that hat last until Spring, and appropriate the amount saved to helping the destitute?" This need not be enlarged upon; any intelligent mind will see at a single glance what magnificent results would follow the general adoption of the course proposed. Is a motive needed? Why, look at it! When at the Vienna Exhibition, last Summer, the largest by many times, the grandest, the most magnificent, by far, the world ever saw, there was an instinctive desire to raise the hat and make obeisance to every passing workman, with his soiled hands, bared arms, and begrimed face, and homely dress; and under it all was a sturdy, honest, manly, independent look, as much as to say, "There is my handiwork; I polished that diamond that a million could not buy; I constructed that engine, the most perfect and powerful in the world ; I invented that lace loom, which will do more work in an hour than fifty women could do in a week!" And so whole pages of contrivances could be enumerated, each one born of the human intellect; each one adding to the comfort, happiness and well-being of mankind, and every one of them the product of the " mechanic's " brain, and the skill of his fingers, and the strength of his own right arm ; and from these the rich have gathered their riches. And when this stalwart and begrimed mechanic is the creator of our wealth, by taking a pound of iron which costs ten cents and makes it into watch springs, selling for a thousand dollars or more, a very small matter it is that we should feed, and clothe, and shelter him comfortably through the whole winter, and longer, if need be, when, from no fault of his own, hard times have come and have snatched the bread from his table and his little ones cry for food. These are the men who had rather starve than resort to crime and live. Where is the monied man who will dare to say, " It's nothng to me; let him help himself." Such a man ought to be considered a discredit to his kind. But, in spite of all that has been written, the man who, in times like these, gives a single penny in charity on the street, or a slice of bread at his door, or allows any of his household to do so, wrongs himself, wrongs the poor, wrongs the community in which he lives, because everybody knows that the least deserving are the very first to go a begging, while the meritorious poor, with a commendable and innate pride, pine in uncommunicated destitution, almost to death's door, before they will ask for charity.

A greater reason still, where so many have to be helped, and there is no knowing how long it will last, the commonest prudence dictates that the means of the charitable should be husbanded with the utmost care, and not a mite of money or an ounce of food should be wasted, but that every thing should be appropriated where it would go the farthest and where it was most needed and was most deserved. Within a week half a loaf of bread in slices was seen in the gutter, thrown there by a person who had gone from door to door with a basket, which, being too full, the bread was thrown away to make room for a large piece of meat, given by servants whose money did not pay for it; and from daylight until ten o'clock at night such things are transpiring every hour in almost every street in New York; and shame, a burning shame, be upon every wife whose guilty indifference to her husband's resources will allow such a thing to be possible at her kitchen door from this hour.

Mr. Hartley, No. 59 Bible House, is at the head of an organization which provides tickets, intended to be kept in every house, and to be carried in every pocket-book, and the plan has worked admirably for many years. When charity is asked, no matter how urgent it may seem to be, give the applicant a ticket, on which is the name, and street, and number of the house; on presenting the ticket there, a person is in waiting who will go to the residence of the individual giving the ticket, and if, upon minute inquiry, the applicant is really in need, then assistance is given—not in money, but in food or house rent. Clothing is distributed with the utmost caution, because it may be easily sold and squandered, and the person receiving a coat to-day, costing several dollars, may be as destitute to-morrow as before it was bestowed upon him. So systematically are these things done that one dollar will give seven persons a comfortable night's lodging, or will supply a full, nutritious meal to ten. Hence, if any good citizen has food, or clothing, or money to give, and desires the most judicious and economical use to be made of it, by all means send it to Hugh

Camp, Esq., 155 Worth street, New York; to the "Ladies Protective Union," 168 West Forty-fifth street; to the "Sisters of the Strangers," No. 4 Winthorp place, Greene street; to Wm. C. Gilman, Esq., 46 Pine street, for the Prison Association, to help discharged prisoners to live until they can get places for an honest living; to the Sick Children's Fund, to that noblest of men Charles L. Brace, Secretary, No. 19 East Fourth street, near Lafayette place; or to any of the Sisters of "Charity" or "Mercy," who, like angels of blessedness, are going about everywhere, and always helping the helpless, and nursing the sick, and caring for all who are in want. If you have money, deposit it with Mr. Hartley and get a handful of the tickets above referred to, and you "will be blessed in your deed." And so, if you have any cast-off clothing, from an old shoe to a night-cap, it will be most thankfully received by Mrs. William H. Dunning, at her residence, 154 West Sixteenth street, and be appropriated with great judgment and conscientiousness to the uses of the poor and needy, for whose benefit she has for years past devoted a large portion of her time, and finds her reward in the appreciation of all who know her.

STRAITENED CIRCUMSTANCES.

There are very many who, although not needing outside aid, yet are of limited means, and these owe a duty to themselves and to the general public, also, to husband their resources at once, in such a way as to make the least money purchase at the lowest price that kind of food which contains the most nutriment, because no one knows how long these hard times will last—and many who do not dream of such a thing now, and may not think it possible, may become a public charge.

Then there is another and a large class of workers, tens of thousands, in every part of the country, who are deceiving themselves, and are pursuing, literally, a most suicidal policy, acting under the influence of a false pride and a false logic, in refusing to work for a smaller price, when they cannot get as much as they think they ought to have, forgetting that, as there is not work for half the workers, it is better to work for a quarter of their usual wages than not work at all—for no work to-day means nothing to eat to-morrow, nothing for themselves, nothing for their wives and children. They talk of their honor in abiding by the rules of their societies, and feel a kind of pride in standing bravely up to their obligations, meanwhile consuming their own resources, to fall back on the contributions of strangers, which means nothing more nor less than living off of the bounty of others, which is beggary, all but the solicitation. But when the funds of guilds give out, as certainly they must do, then comes beggary outright or the poorhouse, and pride and honor both go to the winds together. In times like these it is better for a man to work for his food only, than not to work at all, then his wife and children can have divided among themselves what he would have eaten.

The CHRISTIAN WEEKLY says: "By eating cheap and nutritious food many may save themselves the mortification of becoming a public charge, and to give that information is one of the chief aims of this article. All nutriment contains two elements, one which warms, called carbonates, answering to charcoal, which, when it is burned, gives out heat. Without warmth we die. That warmth is generated by the stove within us; that which feeds the stove, supplies the fuel, is whatever is oily, whatever is sweet. Sweet oil, sugar, butter, are the representatives of this class of food or nutriment, and are almost entirely nutritious. In a hundred parts of oil there are near a hundred parts of carbon or warmth, the same with sugar. Hence, in cold weather we have a greater relish for fats and sweets.

"A Greenlander will take at one meal five pounds of tallow candles and as much hog's lard, butter, or any kind of oil he can get; he needs it to generate warmth enough within to keep him comfortable. But there is as much nutriment of this kind in a pound of lard, costing twenty cents, as there is in a pound of butter at sixty cents. A pound of lard, made into gravy, taken with bread, goes every bit as far as a pound of butter spread on bread—a clear saving of two thirds.

"The other element of food is called the 'nitrates,' making flesh and imparting strength to work. Among these are meats of all kinds, hence the carbonates warm and the nitrates strengthen; a pure carbonate, as oil and sugar, gives no strength—warmth only—and without other food, containing nitrates, a man would soon die. But Beneficent Wisdom has given both constituents to almost all the common articles of food—not only the nutriment of warmth but the nutriment of strength; hence, when it is said in the tables below that one article of food has a certain percentage of nutriment, and another has a different per centage, it means of warmth and nutriment together, but with varying proportions. Beef, for example, has 35 per cent. of nutriment, of which 19 per cent. gives strength, and 14 per cent. of warmth, and 2 per cent. of brain feeding material, called phosphates. Common rice has 92 per cent. of nutriment, eight per cent. being water. In a pound of beef there is 68 per cent. of water. There is as much nutriment in one pound of rice, costing ten cents, as there is in three pounds of beef, costing a dollar. The estimate in the table is for beef without any bone, but the bone has to be paid for. Bacon is cheaper than beef, and goes twice as far; it has only 14 per cent. of water, beef 68 per cent. White beans have 67 per cent. of nutriment, and contain twice as much nutriment as beef, costing, pound for pound, not a quarter as much, making a difference altogether of several hundred per cent.

"In Scotland whole families of working people make their entire breakfast on oat meal porridge, costing but a few cents. Wheaten grits, crushed wheat, Graham bread have all the elements needed to give warmth and strength to the body, and would keep it in health and vigor if nothing else were eaten for weeks and months together. In this way the cost of eating could be easily and healthfully reduced to twenty-five cents a day, and even less, and the person never know the pangs of hunger.

"Very many persons in Ireland live mainly on potatoes, which have but twenty-five per cent. of nutriment, the remainder being water, and they would make a comparatively cheap diet.

"It has been stated that nearly half the globe is mainly fed on rice—the millions of China and Japan make it their chief article of subsistence.

"If a man sits down and leisurely makes a full meal of potatoes, or boiled rice, or oatmeal porridge, or wheaten grits, or homminy, either one of them seasoned with well made gravy of hog's lard, a little flour, with a proper amount of pepper and salt, he will feel no more hunger half an hour afterwards, will have as much satiety, will have quite as much a feeling of satisfaction as to food, will be as much disinclined to eat, as if he had made a dinner on broiled chicken, roast beef, fried oysters, or porter house steak; all the difference is in the amount of pleasureableness experienced during the short period of its going down the throat; just as a thirsty man will feel quite as free from thirst in five minutes, from drinking a glass of spring water as a glass of soda—the only difference being in pleasureableness during the time of swallowing. The practical question to be considered is, 'Ought I, in these times, give a dollar for a dinner of roast turkey, when the system would be as much nourished by a plainer one, costing not a quarter as much,' the only difference being the greater pleasure during the few minutes of eating. All that is needed is a little self-denial during dinner time to save half a million dollars in the City of New York every day. A true humanity would seem to require, in times of scarcity of food and clothing, in the inclement weather of winter, that both rich and poor should practice a reasonable amount of self-denial; the rich, that they may be able to help the poor more liberally, without oppressing or injuring themselves; and the poor, that they should do all in their power to make their calls on those who are helping them as moderate as possible."

WHO WANTS WORK?

Those who really want work to do ought to be willing to work at whatever price they can get, even if it is merely for their board, for there are many who could afford to give board for wages who could not give any more. There are many who could afford to give half a dollar a day for work, but could not give more, because their circumstances, their income, their sales would not justify it. If a girl or boy over twelve years old could get board wages, then the family would have one person less to feed. It is most unwise, under the circumstances of the times, for any one to refuse to work unless full wages are paid. Many a worthy mechanic, who has a family dependent on him, would willingly work for half price—for what he could get—but is restrained by his obligations to some society of which he is a member. That society may pay him a certain amount at stated periods, but they can only do it as long as their money holds out; but when the treasury is exhausted, what then? The poorhouse, beggary, or worse! Meanwhile, he and his family are fed by the labors of others who are so fortunate as to have work, but who do not know how long that will last, and who have families of their own to support. The actual, undeniable result is, that a stalwart, able bodied laborer is doing nothing from day to day to support himself, but goes three times a day to a fellow laborer, and takes a part of the meat and bread from the wife and children who are sitting at the table. It would seem, therefore, under the distressing circumstancee of the times, that men ought to sever their connection with these hampering societies, and bravely strike out for themselves. It is far better to feel independent of all help at half wages, than to be doing nothing and stand in the position of being helped by others who have nothing to spare—who can barely help themselves and those dependent on them.

The question arises, Ought any family be helped to food, or clothing, or money who has a son or daughter in the house over twelve years of age, who is well, and able to get as much as board for wages? Such person has no claim whatever on the generosity of others, and such a thing ought not to be allowed in any case, unless under very exceptionable circumstances—such, for example, as their services being necessary to the family, to the extent of helping them along, or more than their board is worth.

SOUP KITCHENS

and free lodging houses to all who choose to come, and for an unlimited time, are injurious to the public good, and foster idleness with all its attendant demoralizations. Every time a man receives gratuitous aid he becomes less of a man in his own estimation. At first his manly pride rebels, but that rebellion is less and less demonstrative, until it is all exhausted, and the next stage is that it is rather agreeable, and all manhood is dead from that hour henceforth. The true philosophy of gratuitous aid is to give the least amount possible to meet the exigencies of the case, and do the most possible to place the recipient in a position of self-help, even if at first the mere board is the only compensation; and if such a person is found to be really faithful, willing to do anything, only if he or she can make themselves useful, they will be appreciated very soon, and their wages will be voluntarily increased by their employers. This is a necessary, natural and legitimate result of conscientious fidelity the world over.

WORK FOR BOARD ONLY.

An association might be formed at once for securing the names of all persons, male or female, old or young, who can come with proper recommendations for honesty and respectability, who are willing to work for their board. An intelligent young German was a waiter at the Pearl street House, Cincinnati. After a few days he was asked:

"What wages do you get?"

"My board, sir."

"How is that?"

"It was the best I could do when I engaged myself. I am not without money, but I preferred to work for my board only to taking that much money out of my pocket."

"How long will you remain here on these terms?"

"Until I can better myself."

In two years from that time he was an equal partner in a dry goods house on Fifth street, north side. This is the true philosophy, and if all unemployed persons, who come properly recommended, were willing to work for their board, the immediate effect would be that many families, who are about dismissing one or more servants, because they have not the means to pay wages, would gladly employ them. Thousands of other families, who cannot any longer pay twelve or fifteen dollars a month for help, are able to pay five or six, and would do so as soon as the new help deserved it. It is true that thousands of girls now getting high wages would be dismissed, and as many would be out of employment as now are, but this is the difference: The question with many a housekeeper is, Shall I pay twelve dollars a month or shall I do my own work, and, as I have not the money, cannot get it, and have no prospect of getting it, I must do my own work. So the point is whether two persons shall be idle or only one.

Besides, if our richest merchants are sacrificing twenty and thirty per cent. on their goods to adapt themselves to the emergencies of the times, there is no good reason why our servants should not bear their portion of the burdens of the situation, and diminish their wages one fourth or more. If the mistress is to make all the sacrifices and practice all the self-denials, and the maid does nothing of the kind, it is surely high time for the sharp action of a prompt dismissal.

Many a family could do without an "up stairs" girl were it not for the bell calls, and occasional errands not possible to the cook; these things a girl or boy of twelve could easily do, and get their board, and in many cases something over.

### SEAMSTRESSES.

A thousand seamstresses, who are at this moment doing nothing, and are consuming their scanty resources, or living in garrets on a very scarce allowance of food, could find employment in twenty-four hours on the principles above alluded to and have cosy rooms and an abundant table.

### WASHERWOMEN

who now have but an occasional day's work, would be engaged every day of the week for their board and a few cents over, for that would be in many a housekeeper's power, when a dollar a day would be simply impracticable; not that they are not worth a dollar, but they cannot get it, and, if they had it, could not spare it all for that purpose, but might spare a part of it.

Mechanics should bear in mind that they are dismissed, or put on short time, or are docked in their wages a certain percentage, not from any unfriendly feeling on the part of their employers, but from urgent and most imperative reasons; reasons which are perfectly overwhelming and irresistible. First, they cannot collect the money due them for sales of goods and wares already made; second, their customers, seeing the impossibility of getting money, are not willing to incur obligations by purchasing on credit, even at a large reduction of price; third, the people have not the money, nor can they get it, except in small amounts, hence, spend it only for such articles as they cannot possibly do without, and this extends to every department of trade and reaches all conditions and callings in society. And for manufacturers to make goods only to pile up in their warehouses, and for capitalists to erect buildings which cannot be rented, would be perfectly suicidal; disastrous failure would come on apace, and they themselves would be without food or shelter. Hence all persons, the working people especially, must be content for the present to work for what they can get, and to learn to live on the kinds of food which go the farthest and which cost the least money, and which, although they are not as

TOOTHSOME

as more costly kinds, are, beyond all question, more healthful and more invigorating. Hence the tables following, taken by permission from the two books entitled "Health by Good Living" and "Health at Home," can be studied to very great advantage, and would reduce the cost of feeding the people about two thirds. As before stated, the nutriment contained in the ordinary articles of food has two elements, the carbonates, which warm, and the nitrates, which strengthen—that is, make blood and flesh; the proportions of some of them are as follows:

| Name. | Proportion of Carbonates. | Nitrates. |
|---|---|---|
| Arrowroot | 36 | .. |
| Apples | 45 | .. |
| Beef, Roast | 53 | 15 |
| Beans | 88 | 38 |
| Butter | 65 | .. |
| Lard Oil | 80 | .. |
| Lentils | 37 | 38 |
| Lean meat | 13 | 15 |
| Lean and fat meat | 22 | 18 |
| Milk | 10 | .. |
| Olive Oil | 77 | .. |
| Oats | 40 | 2 |
| Peas, dried | 36 | 39 |
| Potatoes | 11 | .. |
| Roast beef | 53 | 15 |
| Rye | 38 | 1 |
| Sweet Almond Oil | 77 | .. |
| Starch | 37 | .. |
| Suet | 79 | .. |
| Sugar | 42 | .. |
| Turnips | 3 | .. |
| Veal | 52 | 14 |
| Venison | 53 | 15 |
| Wheat | 39 | 2 |

If we are chilly, food must be used having the most carbonates; if strength to work is required, then take those articles which most abound in the nitrates.

But the table which is more generally useful, is that which gives the amount of nutriment contained in the ordinary articles of food, as they are found in the markets and on our tables. And, by comparing these with prices, any one can determine what kinds are most economical:

| Kind of Food. | Preparation. | Per cent. of Nutriment. | Time of Digestion. H. M. |
|---|---|---|---|
| Apples | Raw | 10 | 1 30 |
| Barley | Boiled | 92 | 2 00 |
| Beans, dry | Boiled | 87 | 2 30 |
| Beef | Roast | 26 | 3 30 |
| Bread | Baked | 80 | 3 30 |
| Cabbage | Boiled | 7 | 4 30 |

| Kind of Food. | Preparation. | Per cent. of Nutriment. | Time of Digestion. H. M. |
|---|---|---|---|
| Carrots | Boiled | 10 | 3 15 |
| Cherries | Raw | 25 | 2 00 |
| Chickens | Fricassed | 27 | 2 45 |
| Codfish | Boiled | 21 | 2 00 |
| Cucumbers | Raw | 2 | 3 30 |
| Eggs | Whipped | 13 | 1 30 |
| Flour, Bolted | In bread | 21 | 3 30 |
| Flour, unbolted | In bread | 35 | 3 30 |
| Gooseberries | Raw | 19 | 2 00 |
| Grapes | Raw | 27 | 2 30 |
| Haddock | Boiled | 18 | 2 30 |
| Melons | Raw | 3 | 2 00 |
| Milk | Raw | 7 | 2 15 |
| Mutton | Roast | 30 | 3 15 |
| Oatmeal | Baked | 74 | 3 30 |
| Oils | Raw | 96 | 3 30 |
| Peas, dry | Boiled | 93 | 3 30 |
| Peaches | Raw | 20 | 2 00 |
| Pears | Raw | 10 | 3 30 |
| Plums | Raw | 29 | 2 30 |
| Pork | Roast | 21 | 5 15 |
| Potatoes | Boiled | 13 | 2 30 |
| Rice | Boiled | 88 | 1 00 |
| Rye flour | Baked | 79 | 3 30 |
| Soup, barley | Boiled | 20 | 1 30 |
| Strawberries | Raw | 12 | 2 00 |
| Turnips | Boiled | 4 | 3 50 |
| Veal | Fried | 25 | 4 30 |
| Venison | Broiled | 22 | 1 30 |
| Wheat bread | Baked | 95 | 3 30 |

The following table gives the proportion of nutriment and the proportion of fuel in a given quantity of food :

Milk contains one proportion of nutriment, 2 of fuel.
Beans contain one proportion of nutriment, 2½ of fuel.
Oatmeal contains one proportion of nutriment, 5 of fuel.
Barley contains one proportion of nutriment, 7 of fuel.
Wheat contains one proportion of nutriment, 8 of fuel.
Potatoes contain one proportion of nutriment, 9 of fuel.
Rice contains one proportion of nutriment, 10 of fuel.
Arrowroot contains one proportion of nutriment, 26 of fuel.
Tapioca contains one proportion of nutriment, 26 of fuel.
Sago contains one proportion of nutriment, 26 of fuel.
Starch contains one proportion of nutriment, 40 of fuel.

The last named articles are given to young children, because they require a great deal of warmth. But they need more than warmth; if fed on these alone, they would soon die; hence milk must be added to these, as it contains materials for growth and repair.

In general, the breads and other preparations of grains have double the nutriment contained in meats, pound for pound.

The above tables are the result of the latest scientific investigations, and are sufficiently near the truth to answer all practical purposes as a general rule, there being individual exceptions.

To ascertain the relative value of any article of food the amount of nutriment in a given quantity must be considered in reference to its cost. There are four pounds of nutriment in a hundred pounds of turnips, and thirteen in potatoes. If three pounds or bushels of turnips cost more than one pound or bushel of potatoes, then the latter is a cheaper food.

The CHRISTIAN WEEKLY says: "The explicit rule of diet for those whose circumstances are limited: Breakfast—oatmeal porridge, cracked wheat or hominy, alternaitng them and using salt, or sometimes sugar, syrup, treacle or milk. Dinner—meat with potatoes, turnips, onions or beans, with corn bread or Graham, nothing else whatever—no dessert. If drink is needed take half a glass of cold water, or which is better, a cup of hot drink; the best is milk and water, half and half, sweetened or not. Drink no cold water within an hour after a meal. Let any one, sedentary or laborious, try such a diet for a week, and, other things being equal, he will weigh more, be stronger, and be in better health than before, but he should weigh himself at the same hour of the day, in the same clothing and on the same scales. If in the ways suggested, the 'well-to-do' will put their shoulders to the wheel and curtail some of their luxuries, and those who are not so 'well off' will do their part, by practising their share of self-denial and use less costly food, although quite as nutritious and healthful as that eaten in better times, the rich and the poor, laboring side by side in the generous aims of mutual help, all will think the better of themselves, and the happier will they be in the very near future, before the daisy peeps its little cheering head above the snow, when the 'flush times' come, as come they must and will, as inevitably as the coming of the joyous Spring, for the simple reason that the laws of trade will compel the result. We have three or four hundred millions of grain to sell, the continental nations will have a famine unless they purchase large quantities of it, they have the gold to pay for what they buy, and must purchase or starve by tens of thousands. Hence, amid the darkness of the present hour, there's a

'Light in the window'

for us all, and unequalled prosperity in the early spring."

